# Origin and Persistence of *Lycopodium clavatum* and *Lycopodium annotinum* (Lycopodiaceae) in Scots Pine Forests

**DOI:** 10.3390/plants13152120

**Published:** 2024-07-31

**Authors:** Radvilė Rimgailė-Voicik, Aleksandras Voicikas, Julija Fediajevaitė, Sigitas Juzėnas, Jolanta Patamsytė

**Affiliations:** 1 Life Sciences Center, Institute of Biosciences, Vilnius University, Saulėtekio Ave. 7, 10257 Vilnius, Lithuaniasigitas.juzenas@gf.vu.lt (S.J.);; 2 School of Biological Sciences, University of Reading, Whiteknights, Reading RG6 6UR, UK

**Keywords:** clonal growth, club mosses, ISSR, small-scale disturbance

## Abstract

Understanding the growth dynamics of spore-bearing clonal plant sporophytes and the influence of abiotic and biotic factors is crucial for predicting the persistence of club moss populations and implementing effective habitat management techniques. Despite this, the longevity and development of club-moss populations are rarely studied. This study adopted an integrated approach to assess the probability of repetitive young sporophyte recruitment via sexual propagation in *Lycopodium annotinum* L. and *Lycopodium clavatum* L. The size–age problem of clonal spore-bearing forest plants and their niche segregation were addressed. The canopy characteristics, insolation, small-scale disturbance, and genetic polymorphism were studied in temperate semi-natural Scots pine forests in Lithuania. Based on the size of the clones discovered, we hypothesize that initial sporophyte emergence occurred in 20-year-old pine stands, with subsequent sporophyte emergence continuing over time. The emergence was related to small-scale disturbances. High genetic polymorphism indicates that all sporophyte stands studied likely emerged via sexual reproduction. According to Ellenberg values, *L. annotinum* is related to shady habitats, but our findings show both species coexisting abundantly in the more open habitat, supposedly more suitable for *L. clavatum*.No significant differences in vegetation relevés and light availability was detected using hemispheric images.

## 1. Introduction

The balance between sexual recruitment and clonal offspring production is not well understood in land plants, and general explanations for low rates of seedling recruitment and clonality often point to malfunctions in sexual reproduction, such as seed or partner deficiency, trade-offs between clonal and sexual reproductive allocations [1], or environmental restrictions [2]. In woodlands, plants exhibit escape mechanisms to exploit spatially unpredictable patches, making them less dependent on seasonal regeneration [3]. Seedling establishment is less common compared to grasslands, and forest-floor species are usually adapted to long-distance dispersal [4]. Club mosses, in particular, exhibit low competitiveness [5,6] and tend to respond to unfavorable disturbances by growing away from such areas [7].

The life cycle of *Lycopodium* consists of two separate generations: gametophytes (n) develop in the soil, while sporophytes (2n) are present on the forest floor. Sexually, club-moss sporophytes originate from subterranean, achlorophyllous gametophytes which require a specific group of endophytic fungi to form [8,9]. Recent studies have demonstrated that successfully developing gametophytes are colonized by Densosporaceae (Endogonales and Mucoromycotina) fungi [10]. Continuous vegetative propagation likely enables club-moss sporophytes’ persistence despite low or no sexual reproduction, as the genet turnover rate is very slow. Clonal areas typically form from clumped and intermingled groups of genets and ramets. The club moss’s outgrowth formation and percentage of genets and ramets in a single outgrowth, and their spatial distribution patterns remain unknown. Physiological ramet integration allows for acropetal and basipetal resource translocations, as demonstrated in arctic [11] and temperate deciduous forest [12,13] clonal plants.

The genome size evolution and the degree of plasticity in long-lived clonal archaic spore-bearing vascular plants are of considerable biological interest, but they remain poorly understood [14,15]. Based on the frequency of sexual recruitment, all clonal plants can be separated into two major groups: (a) populations with repeated regular seedling recruitment and (b) populations with initial seedling recruitment and rare seedling addition [16]. It is unclear to which one lycophytes should be assigned due to the wide life-cycle timeframe and the rarity of gametophyte discovery [17]. Juvenile club-moss sporophytes are spatially isolated from mature *Lycopodium annotinum* L. and *Lycopodium clavatum* L. plants [8,18], which tend to form large outgrowths [5,7,19].

In clonal plants, mother plants produce ramets only in neighboring patches, and their performance is often regulated by local density rather than the whole population density. The persistence of genets in natural clonal populations largely depends on meristem demography [20]. It is believed that plant roots originated independently in lycophytes and euphyllophytes, but the processes of root evolution remain contentious, and little is known about the parallel evolution and molecular mechanisms governing lycophyte root functions [21]. It is generally accepted that Lycopod roots evolved around 400 million years ago and lack both the vascular cambium and organogenic properties outside the apical meristem [22]. Mature club moss sporophytes are opportunistic foragers that are well adapted to patchy nutrient resources [5,23]. The frequency of club-moss sexual reproduction is debatable and may depend on the level of habitat disturbance. Previously, club moss subterranean gametophytes and juvenile sporophytes were found on and near forest roads and tracks, near lines separating forest blocks and skiing tracks [8,17,24]. The presence of fire adaptations is also expected [19,25].

**Figure 1:**
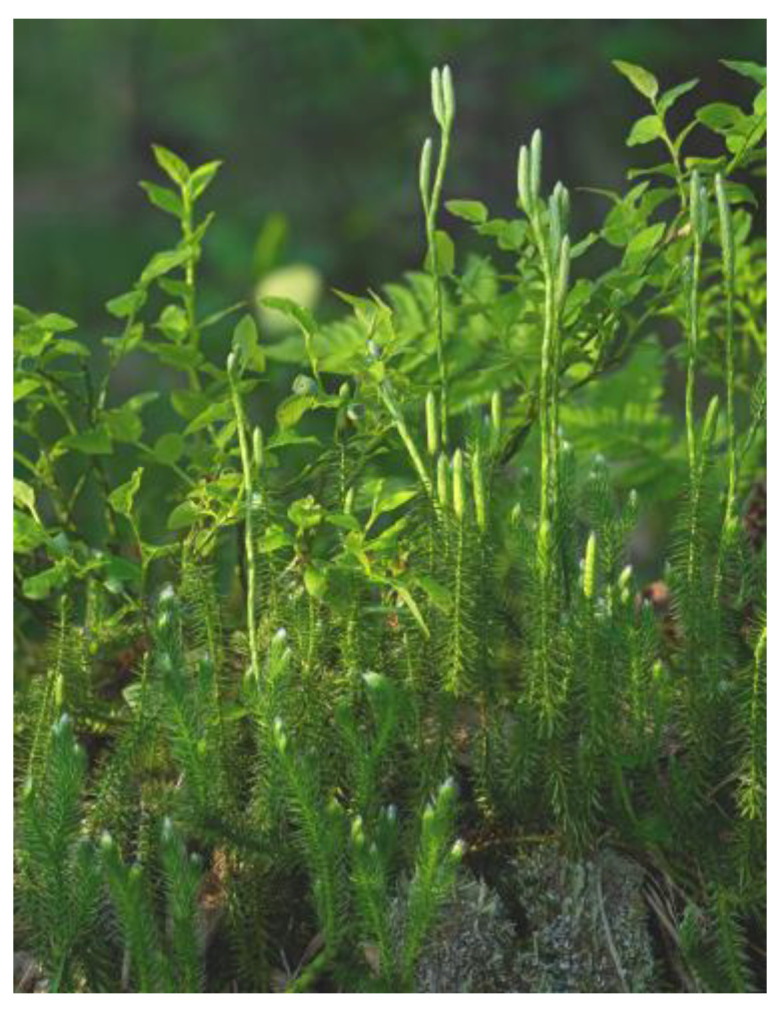
*Lycopodium annotinum* and *Lycopodium clavatum* forming an intermixed outgrowth in Verkiai Regional Park, Vilnius city, Lithuania. Photo by Greta Valvonytė.

*Lycopodium annotinum* and *L. clavatum* (Figure 1) are crucial forest-floor species that are characteristic of the protected Boreal Europe Forest type Western Taiga (code 9010) [26]. Members of the *Lycopodium* genus are protected and considered to be of community interest. Their collection from the wild and exploitation may be subject to specific management measures at the national level within European Union member states [27]. Additionally, *L. annotinum* is listed on the International Union for Conservation of Nature’s (IUCN) Red List of Threatened Species, classified as being of ‘least concern’ in Europe [28]. Live specimens of *L. clavatum*, as well as dried and fresh plants, including leaves, roots/rootstocks, stems, seeds/spores, bark, and fruits, are subject to Annex D of the EU Wildlife Trade Regulation 318-2008 [29].

Stable understory vegetation species groups have been widely used in the major forest-habitat classifications [26,30] and the Ellenberg indicator value system (EIVs) [31,32]. Understory vegetation significantly influences the composition of future forests in response to disturbance [33] and plays a crucial role in nutrient cycling within forests [34]. Yet, there is still no universal agreement upon the concept of forest health with the respect to the whole forest ecosystem [35].

Club mosses are an important forest-floor component with significant indicator value for forest health assessment. It is unclear whether the origination of new club moss sporophytes is related to higher levels of forest floor disturbance during logging or forest clearing, or if it is linked to repetitive moderate to low disturbances. The impact of disturbance on successful club moss sporophyte emergence through sexual reproduction and maturation was never tested. In general, the life history and population dynamics of forest species are strongly shaped by timber extraction activities, which can disrupt the long-term resilience of forests and reduce the delivery of ecosystem services [36]. We hypothesize that the origination of club moss sporophytes from gametophytes is more closely associated with small-scale disturbances than with one-time catastrophic events such as forest fires or clear-cuts.

In this article, we compare *L. annotinum* and *L. clavatum* in Scots pine stands based on sporophyte outgrowth size, surrounding vegetation, light availability, and genetic polymorphism. The selected methods were not invasive and ensured low-to-no damage to club moss stands. This research highlights the importance of understanding the adaptive strategies of club moss species, which can inform forest management practices and conservation efforts. Further studies are needed to explore the long-term impacts of environmental disturbances on club moss populations and their role in forest ecosystem health.

## 2. Results

Lithuanian forest cadastral data showed that trees in researched sites in Vilnius County were of similar age, with an average age of 91 ± 18 years, ranging from 52 to 127 years. The tallest trees were 24–28 m in height. The tree stands in all sites consisted of *Pinus sylvestris* L., with no subdominant or shrub layer being present. The most common understory species were *Vaccinium vitis-idaea* L., *V. myrtillus* L., and *Melampyrum pratense* L. The moss cover was almost 100%, consisting mostly of *Pleurozium schreberi* (Brid.) Mitt., *Hylocomium splendens* (Hedw.) Schimp., and *Dicranum* Hedw. sp. We compared vegetation relevés of mature *L. annotinum* and *L. clavatum* sites (Veržuva, Nemenčinė and Sakiškės) with juvenile *Lycopodium* growth sites (Varėna) relevés. Reyni diversity profiles were generated for forest sites, and ANOVA revealed significant effects of log species’ richness (α = 0), F (90) = 3.16, *p* < 0.03. A post hoc comparisons showed that the Sakiškės site differed from the Varėna site (Tukey HSD, *p* < 0.01). No significant differences among forest sites were found in Shannon (α = 1) and Simpson (α = 2). A *t*-test also showed that mature forests differ from juvenile forests in Varėna significantly (t (92) = 2.7, *p* < 0.01, d = 0.7). ANOVA revealed significant differences among forest sites in the proportion of the most abundant species ((α = Inf), F (3, 90) = 4.8, *p* < 0.03). The post hoc analysis showed that Varėna differed significantly both from Nemenčinė and Sakiškės (*p* < 0.01). Additionally, sites with mature sporophytes differed significantly from sites with juvenile sporophytes: t (92) = 3, *p* < 0.003, d = 0.8.

**Figure 2:**
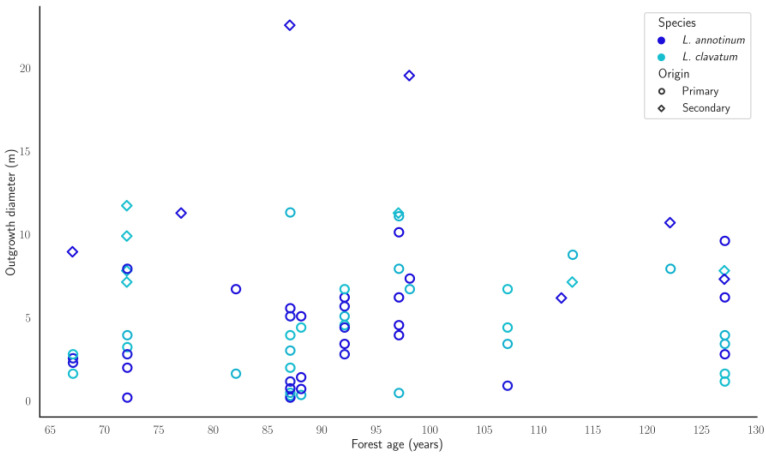
Scatter plot of forest age and club moss outgrowth diameter. *L. annotinum*—*Lycopodium annotinum*; *L. clavatum*—*Lycopodium clavatum*. Primary clones, marked with dots, are circular or partially circular and most likely originated from gametophytes. Secondary clones, marked with diamonds, are differently shaped, and their origin is unclear.

No significant correlation between forest stand age and clone size was found (Figure 2). According to the outgrowth size, the latest emergence of *L. annotinum* happened when the forest stand was 110 years of age, and for *L. clavatum*, when the stand was 100 years old. The age of sporophytes varied greatly, with a plausible 20-year gap between youngest and oldest sporophyte. Based on yearly growth rates, the age dispersion of *L. annotinum* sporophytes was 45 years, and for *L. clavatum*, it was 40 years. *Lycopodium annotinum* and *L. clavatum* populations emerged when trees were around 20 years of age, and population enrichment with new juvenile sporophytes was repetitive.

Weighted Ellenberg indicator values calculated for the grass–subshrub layer were used to test for differences among forest sites. ANOVA revealed a significant effect among forest sites based on species preference in temperature value (F (3, 90) = 3.14, *p* < 0.03). The post hoc analysis indicated a significant difference between the Nemenčinė and Varėna sites with juvenile sporophytes (Tukey HSD, *p* < 0.02). Additionally, the *t*-tests showed significant differences in temperature value (t (92) = 2.6, *p* < 0.01, d = 0.7) and in nutrients value (t (92) = 2.22, *p* < 0.03, d = 0.6) when comparing forests with mature sporophytes to those with juvenile sporophytes in Varėna.

**Figure 3:**
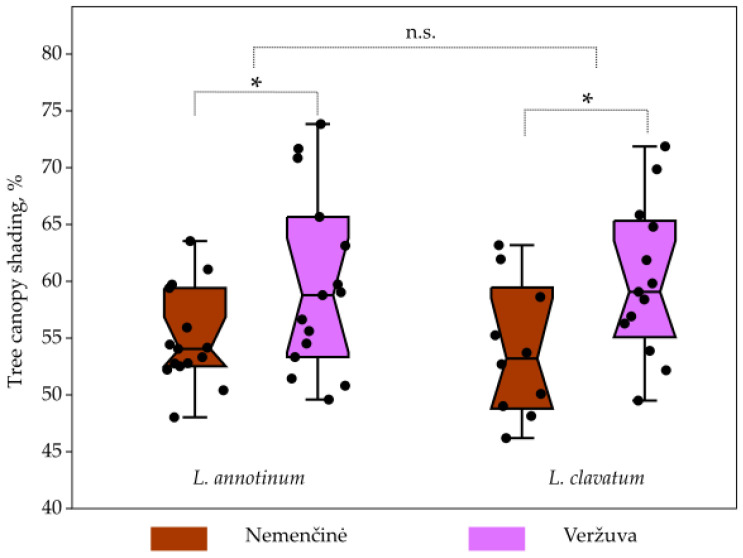
Tree canopy shading in Nemenčinė and Veržuva sites for *L. annotinum* and *L. clavatum* sporophytes. Statistically significant *p*-values, indicated by asterisks (*), and non-significant *p*-values, indicated by ‘n.s.’ (not significant), were determined using a two-way ANOVA.

In the Nemenčinė and Veržuva sites with *L. annotinum* stands, overall tree canopy shading ranged from 48 to 74%, and in sites with *L. clavatum*, shading ranged from 49 to 70%. The variation coefficient for *L. annotinum* sites was 12.5%, and for *L. clavatum* sites, it was 12.6%; thus, no statistically significant tree canopy shading differences were determined (Figure 3).

Also, within the outgrowths in the Nemenčinė and Veržuva sites, the determined vegetative shoot cover in 1 m^2^ was similar and varied from 5 to 50% both in *L. annotinum* and *L. clavatum* (Table 1). The average number of strobili in *L. annotinum* was 10.9 ± 10.3, and in *L. clavatum*, it was 7 ± 7.1. The maximum number of strobili in *L. clavatum* was 1077 and in *L. annotinum* was 2218.

The size of club moss outgrowths varied greatly, but no significant differences were determined between the species. In Nemenčinė and Veržuva sites, the smallest outgrowth of *L. annotinum* was 0.02 m^2^, and the largest was 300 m^2^, with an average of 35 m^2^ ± 50.8 (n = 45). For *L. clavatum*, the smallest outgrowth was 0.6 m^2^, and the largest was 400 m^2^, with an average of 38.9 m^2^ ± 73 (n = 31).

Within a ten-meter radius around the *L. clavatum* and *L. annotinum* outgrowths, no excessive forest damage was observed; specifically, no stumps, tree cutting, windthrow, or fire marks were found. Only forest paths with moss or grass cover were present near or went through the club-moss stands. The impact of forest paths on the horizontal *L. annotinum* and *L. clavatum* clone structure was noticed. The width of the path positively affected the development of young shoots and mature and dried-out shoots and negatively correlated with fertile shoots in *L. annotinum*. In *L. clavatum*, path width also positively correlated with young shoots and negatively with mature and dried-out shoots. Both club-moss species reacted to forest road-usage intensity by reducing their sporulation intensity.

**Figure 4:**
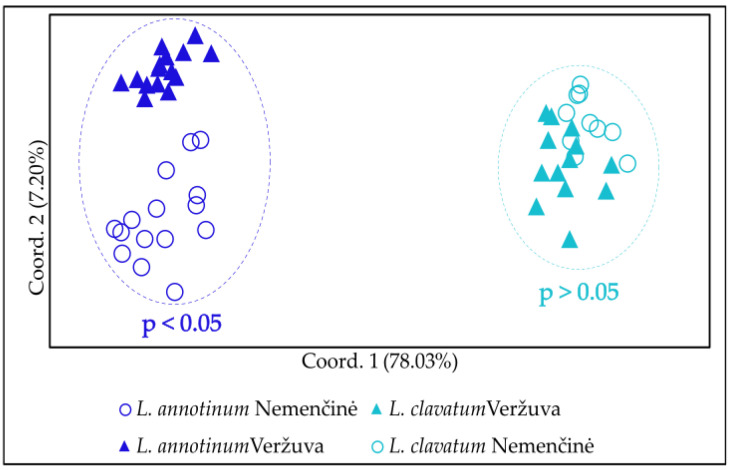
Principal coordinates analysis (PCoA) reflecting differentiation among sporophytes of *Lycopodium annotinum* and *L. clavatum*, using ISSR method, in Veržuva and Nemenčinė. Results of PERMANOVA test using Nei genetic distances for each species provided. *L. annotinum* n = 14 in Veržuva and n = 15 in Nemenčinė. *L. clavatum* n = 13 in Veržuva and n = 10 in Nemenčinė.

The level of polymorphism differed between *L. annotinum* and *L. clavatum* species (Table A1). The polymorphism in *L. clavatum* was higher (74.21%) than in *L. annotinum* (68.02%). Nei genetic distances [37] differed significantly (PERMANOVA, F = 1.16, *p* < 0.05) among *L. annotinum* subpopulations in the Veržuva and Nemenčinė sites, while *L. clavatum* overlapped (Figure 4). The Mantel test showed a positive significant correlation (r = 0.582, *p* < 0.05) among Nei distances and geographical coordinates of *L. annotinum* clones, while the *L. clavatum* results were insignificant.

We conclude that, in the researched sites, mature *L. annotinum* and *L. clavatum* sporophytes occupy complementary niches, thriving in habitats characterized by similar species diversity, recurrent minor forest floor disturbances, and nearly identical light conditions. The ISSR analysis revealed that all outgrowths researched were not identical and, therefore, are a result of club moss sexual propagation. The results presented support the hypothesis that the origination of club moss sporophytes from gametophytes is more closely associated with understory vegetation diversity and small-scale disturbances.

## 3. Discussion

The balance between sexual and vegetative propagation is a key evolutionary feature of the life history strategies among clonal plants. Clonal spore-bearing plants are vital components of temperate forests, and yet few studies have addressed club moss sporophyte stands, their propagation strategy, and the abiotic and biotic factors influencing their successful sexual propagation. Bruchmann [8] was able to locate club moss gametophytes and juvenile sporophytes only in spruce plantations of 10–20 years of age and argued that only one age class, related to the time of forest planting, can be determined. Our findings align with those of other researchers [19,38], indicating that the emergence of new club moss sporophytes on the forest floor is linked to small-scale disturbances. The absence of these small-scale disturbances, along with interspecific competition, is the primary factor limiting the successful sexual propagation in club mosses.

Only a few studies have addressed the age–size problem in *Lycopodium* species (Table A2). Wittig [6] suggested that if growth rates during the past hundred years have remained unchanged, the average age of the population can be predicted quite accurately. Oinonen [39,40] constructed a timetable of vegetative spreading for woodland clonal species from parallel measurements and used it to determine plant emergence dates and their relationship with forest floor fires, other clonal growth species, and the age of the forest stand. However, as Oinonen emphasized, it has been difficult to determine the origin of the club-moss outgrowth confidently. After a fire, sporophytes can emerge from gametophytes or develop through vegetative spreading from relict plants that survived. Following partial destruction, new juvenile sporophytes can develop and form intermixed outgrowths [39]. Additionally, *L. clavatum* is known to form fairy rings, which are associated with nutrient withdrawal from the soil [41]. 

According to Oinonen [19], vegetative spreading of *L. clavatum* at normal speed begins after 21–22 years, while for *L. annotinum*, it starts after 17–18 years. Plagiotropic shoots survive longer than orthotropic shoots and begin to sporulate after three to seven years [5]. It has been proposed [6] that club mosses reach their maximum growth rates 18 years after the emergence of sporophyte. Considering that approximately eight years pass from spore germination, gametophyte maturation, and fertilization, it may take up to 25 years for *L. clavatum* and *L. annotinum* plants to begin spreading at their maximum potential. Wittig [6] suggested that, in Europe, *L. annotinum* can grow up to 20 cm/year. Yet, the growth rates determined vary (Appendix A Table A2). In Lithuania, the annual growth of young *L. annotinum* sporophytes and growth dynamics in *Vaccinium myrtillosum* Scots pine stands [38] showed that the populations were quite abundant: more than twenty young sporophytes were present and survived during a four-year study, and the average horizontal growth of young sporophytes from 1987 to 1991 was 10.6 cm (Appendix A Table A3).

The relationship among forest stand age, time since fire, and size of *L. annotinum* and *L. clavatum* outgrowths was investigated in Finland [19,39], showing that these factors are related. However, the outgrowth size was more closely related to the time since the last fire than to the age of the forest stand. We were unable to trace the history of forest fires in the study sites, and we found no significant correlation between forest age and sporophyte outgrowth diameter. According to the outgrowth size variation, we conclude that juvenile sporophytes originated from gametophytes repetitively, through different stages of forest development, starting with Scots pine stand of 20 years of age.

According to the Ellenberg values [31,32], *L. annotinum* has a light value of three and grows in sites where insolation is less than five % and vegetation cover exceeds 95%. *Lycopodium clavatum* has a light value of eight and grows in areas where insolation is at least 40% and vegetation cover is not more than 60%. In the dry pine forests of Eastern Lithuania, *L. annotinum* is often found in close proximity to *L. clavatum*, in habitats more suitable for *L. clavatum*. It was demonstrated that *L. annotinum*’s horizontal segment length and number of vertical apices significantly increased under the canopy of *Vaccinium myrtillus* compared with *Vaccinium vitis-idaea*; the canopies differed in regard to their red/far-red ratio, with *V. myrtillus* having the lowest ratio [42], but no similar research is present for *L. clavatum*. During research conducted in Canada, it was observed that three lycophyte species (*L. annotinum*, *L. clavatum*, and *Lycopodium dendroideum* Michx.) exhibited a notable affinity for old clear-cuts (aged 23–54 years) in mixed Acadian forests [43]. These species were evaluated as mid-seral invaders in the region, persisting longer in the community only if light and other understory conditions remained stable.

More than 95% of young sporophytes found in Scots pine forests in the Varėna District were *L. annotinum* [18]. It is plausible that *L. annotinum* exhibit sexual reproduction and, through the gametophytic life-cycle stage, expands the niche, becoming better adapted to environmental pressures related to global warming and anthropogenic contamination than *L. clavatum*. However, the determined polymorphism in *L. clavatum* was higher (74.21%) than in *L. annotinum* (68,.02%). Intensive vegetative propagation is usually associated with genetic monomorphism [44]. This has been shown to be true for *L. annotinum* stand [6] and population of *Lycopodium lucidulum* Michx. [45]. However, other genetic studies of clonal plants [46,47] have opposed this view, showing that populations rarely consist of one or few genets. If intragametophytic selfings were dominant, club moss populations would be expected to have low genetic diversity [48], but this was shown not to be the case [49]. The level of polymorphism discovered suggests that all club-moss sporophytes in the researched territory emerged from gametophytes rather than via vegetative propagation.

Scots pine exhibits high ecological plasticity but typically dominates nutrient-poor, dry sites, forming uneven-aged or cohort-structured stands driven by recurrent surface fires and/or gap-and-patch dynamics. In Europe, these stands are primarily shaped by site-specific disturbance regimes [50]. In Lithuania, clear-cutting accounted for 64% of the total area of felling in the state and private sector in 2021 [51]. The typical management cycle of Lithuanian boreal pine forests involves planting or sowing with soil preparation, two or three thinnings at 20-year intervals, and main (clear or selective) cutting at about 100–170 years of age, depending on the forest group [37]. Recently, management techniques oriented closer to natural forest processes, such as shelterwood cuttings, have been implemented [52]. *Lycopodium clavatum* was the most common species found in all forest types surveyed: clear-cuts, young stands, and mature pine forests; *L. annotinum* were only found in mature pine forests (unpublished research). *Lycopodium clavatum* coverage increases to more than 50% in Scots pine stands of 86–100 years of age (6 plots checked), and the first emergence was reported in the age group of 26–35 years (13 plots checked), with up to 20% coverage (Appendix A Table A4). The whole community-disturbance frequency indicator value for *L. annotinum* is −1.94, suggesting that the return time is nearly a century, while the herb layer disturbance-frequency indicator value is −0.92, indicating adaptations to a small-scale disturbance occurring less than ten years apart [53]. Because of the current forest management policy, forests older than 100 years in Lithuania are rare, and clear-cuts pose a threat to club-moss populations. The main recommendation proposed to keep club-moss sporophytes viable during clear-cut logging is to leave a fragment of tree stand understory [54,55]. Further research is needed to better understand how club mosses are adapting in changing environments and how the origin of club-moss sporophytes influences population vitality and longevity.

## 4. Materials and Methods

### 4.1. Study Area

We conducted assessments of dry pine forests in Nemenčinė and Sakiškės, Vilnius District; and Veržuva, Vilnius City. The overall distance between Nemenčinė and Sakiškės was five kilometers, and between Sakiškės and Veržuva, around eight kilometers. Additionally, sites with juvenile club mosses assessed in Varėna District were incorporated into the analysis.

The Scots pine (*Pinus sylvestris* L.) stands, predominantly of the Vacciniosa and Vaccinio myrtillosa forest types, grow on sandy, nutrient-poor dry podzols and are the most common in Lithuania, covering up to 37% of the total forested area [56]. These oligotrophic pine forests belong to the alliance *Dicrano*-*Pinion sylvestris* (Libbert 1933) W. Matuszkiewicz 1962 of the boreal coniferous forests (class *Vaccinio*-*Piceetea*). 

Overall, the territory can be classified as recent forest (RF), with the age up to two hundred years [57], and has records of agricultural activity, including traditional even-aged (clear-cut) silviculture and salvage logging after natural disturbances. Most pine forests in Southeast Lithuania have a long history of moderate human disturbances and are often visited by locals and holidaymakers, so they are crisscrossed by numerous paths.

The annual gross solar irradiance was similar in all sites, approximately 3300–3400 MJ/m^2^, while the soil surface albedo was about 20% in Vilnius County sites, and in Varėna District it was lower—less than 17.5%. The mean annual temperature in Vilnius is +6–6.5 °C, with an absolute maximum of +34.9 °C and an absolute minimum of −32.2 °C, and the temperature remains above 0 °C for 248 days a year. The mean annual temperature in Varėna District is +6–7 °C, with an absolute maximum of +35.6 °C and an absolute minimum of −35.9 °C, and the temperature remains above 0 °C for 252 days a year. Snow cover is about 20–25 mm and lasts up to 95–100 days per year in Vilnius County and up to 90 days per year in Varėna District. Mean annual precipitation in all sites was 650 mm [58].

### 4.2. Sampling Design

**Figure 5:**
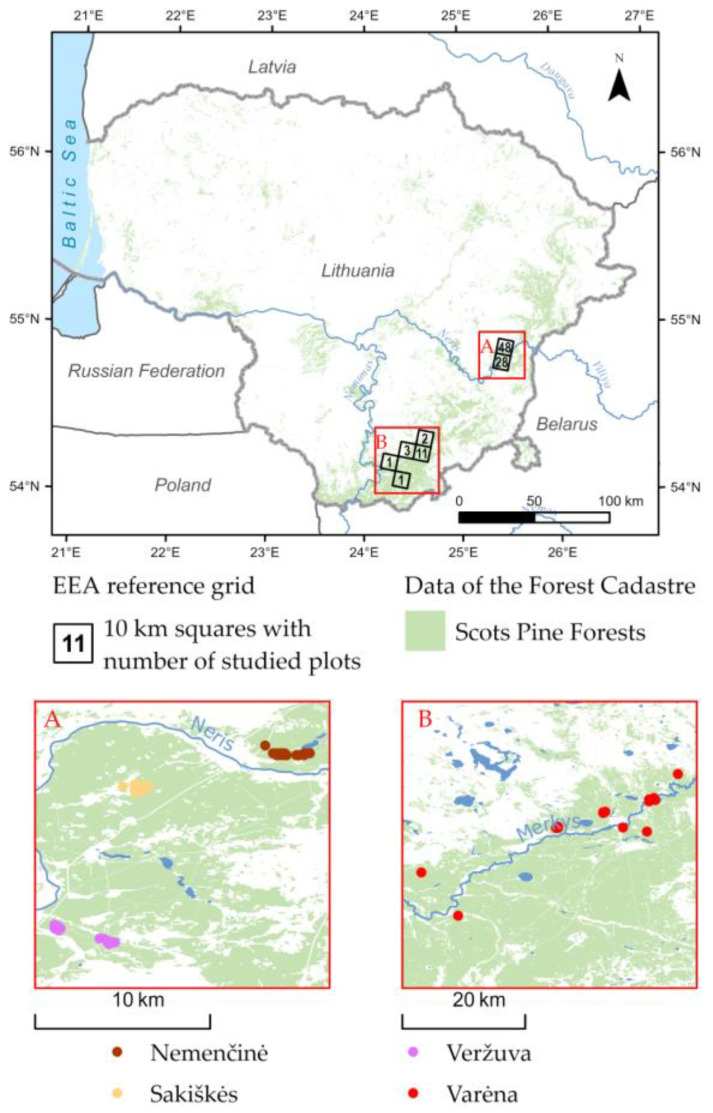
Distribution of club moss sporophyte sampling sites and Scots pine-dominated forest cover in Lithuania.

From July to September in 2016 and 2017, a total of 76 adult club-moss sporophytes were analyzed by RRV and AV in Vilnius District (Supplementary Table S1): 25 in Nemenčinė site (15 *L. annotinum* and 10 *L. clavatum*), 28 in Veržuva site (15 *L. annotinum* and 13 *L. clavatum*), and 23 in Sakiškės site (15 *L. annotinum* and 8 *L. clavatum*). In 2016, sites with club moss outgrowths were identified. At each site, 10 × 10 m vegetation relevés were performed, the approximate size of each clone was evaluated, and samples for genetic analysis were collected. Only detached stands with a distance of 10 m or more between them were selected. Additionally, 18 vegetation relevés with juvenile club-moss sporophytes assessed in Varėna District in 2013–2015 by RRV were incorporated into the analysis (Figure 5). 

We utilized the European Environment Agency (EEA) reference grid, with a resolution of 10 km × 10 km, to illustrate the distribution of club moss sporophyte-sampling sites in Lithuania (Figure 5). Additionally, Figure 5 incorporates detailed data on Scots pine-dominated forest cover in Lithuania, obtained from the Lithuanian Forest Cadastre [59].

In 2017, JF and SJ revisited the outgrowths in the Nemenčinė and Veržuva sites. They assessed the cover of healthy and dried vegetative shoots within the outgrowths, counted the number of strobili, and evaluated the effect of disturbances. For light availability analysis, images were taken at a height of 40 cm from the ground, using a Canon EOS 50D camera with a SIGMA 4.5 mm Circular FISHEYE lens. Hemispheric photos were processed with SideLook 1.1.01 [60] and analyzed with Gap Light Analyzer (GLA) 2.0 [61]. Observations of nearby disturbances, such as paths, pits, and dead trees, were also recorded.

### 4.3. Statistical Analysis

To evaluate and compare diversity, Renyi diversity profiles among the sites were generated in R using picante package [62]. Renyi diversity profiles were generated for the vegetation relevés of forest sites, and ANOVA was employed to test for significant effects, followed by Tukey HSD post hoc comparisons.

Sporophyte outgrowth size and relative age relationship with forest stand age were addressed using regression equations [19,39] and forest-age data. Forest stand-age data were obtained from the Lithuanian Forest Cadastre [59]. 

Regressive equations were formulated using the South Finland forest research data, [19], including a correction of eleven meters for secondary *L. clavatum* stands and eight meters for secondary *L. annotinum* stands:

*d_LC_* = −10.63 + 0.5*a*

*d_LA_* = −7.5 + 0.43*a*,

Later [39], the equations were adapted as follows:

*d_LC_* = −8.175 + 0.455*a*

*d_LA_* = −7.339 + 0.424*a*,

where *d* is the clone diameter, *a* is the age of the sporophyte, *LA* is *L. annotinum*, and *LC* is *L. clavatum*.

Circular or partially circular stands were included in the analysis as primary clones, while differently shaped outgrowths were attributed to the secondary clones, and their length was used as a diameter.

Only well-defined and reproducible DNA bands were included in the binary data matrix. The percentage of polymorphic loci, population genetic differentiation coefficient (GST), and expected heterozygosity were calculated using program POPGENE, version v. 1.31. Principal Coordinates Analysis (PCoA), Permutation AMOVA, and Mantel test based on Nei’s [63] genetic distances were conducted using GenAlEx v. 6.5 [64].

Data processing, statistical analysis, and illustrations were performed in Python, using pandas [65] and pingouin [66] libraries.

### 4.4. DNA Analysis

Outgrowths from Nemenčinė and Veržuva were analyzed. DNA was purified from fresh apical branches of *L. annotinum* and *L. clavatum*, using the modified CTAB method [67]. DNA concentrations were measured using a BioPhotometer, and then samples were frozen and stored at −20 °C. ISSR-PCR analyses were performed [68], and for PCR, we used 5 ng/μL DNA samples. Each PCR amplification was performed in a 48 10 μL reaction mixture (1 μL 10 × Taq MgCl_2_ buffer, 1.2 μL 25 mM MgCl_2_, 1 μL 2 mM dNTPs, 0.4 μL primer, 4.32 μL deionized H_2_O, 0.08 μL Taq DNA polymerase, and 2 μL of club moss DNA). ISSR reactions were carried out in an Eppendorf thermo cycler. The reaction program was set to 94 °C for 7 min, 32 cycles of 94 °C for 30 s, 55/39/46 °C for 5 min, 72 °C 2 min, and a final extension of 72 °C for 7 min. Fourteen primers were used, and seven were chosen for further analysis: ISSR-B, ISSR-C, ISSR I-28, ISSR I-39a, ISSR I-50a, WARD-2, and ARCADE-3. Sequences of these primers are provided in Appendix A, Table A1.

The amplification products were analyzed using electrophoresis in a 1.5% TBE agarose gel. The gel was run for about 3.5 h. Gel results were registered using a BioDocAnalyse documentation system (Biometra, Göttingen, Germany). GeneRulerTM DNA Ladder Mix (100–10,000 bp) was used as standard. 

## Figures and Tables

**Table 1:** Growth data summary of two *Lycopodium* species.

Measurement, m^2^	Result
***Lycopodium annotinum* L. (n = 30)**	
Average vegetative shoot cover	23.9 ± 11.4%
Min vegetative shoot cover	5%
Max vegetative shoot cover	50%
Average dried-out shoot cover	10.1 ± 8.3%
Average number of strobili	10.9 ± 10.3
Total maximum number of strobili	2218
***Lycopodium clavatum* L. (n = 23)**	
Average vegetative shoot cover	17.9 ± 11.3%
Min vegetative shoot cover	6%
Max vegetative shoot cover	50%
Average dried-out shoot cover	10%
Average number of strobili	7 ± 7.1
Total maximum number of strobili	1077

## Data Availability

Data is contained within the article or Supplementary Materials.

## Supplementary Materials

The following supporting information can be downloaded at https://doi.org/10.5281/zenodo.12802736.

## Appendix A

**Table A1:** DNA polymorphism of club moss species determined by ISSR analysis.

Primer	Sequence 5′→3′	Annealing Temperature, °C	Analyzed Bands		Polymorphism, %		DNA Band Length, bp	
			LA	LC	LA	LC	LA	LC
ISSR B	(AG)_8_CG	51	6	7	50.00	57.14	700–1150	600–1250
ISSR C	(AG)_8_TG	49	10	7	50.00	57.14	620–1500	600–1500
ISSR I-28	(GT)_6_CG	39	23	23	78.26	69.57	480–2000	580–2100
ISSR I-39a	(AGC)_4_AC	42	22	21	22.73	38.1	480–2100	580–2500
ISSR I-50a	CCA(GCT)_4_	47	26	23	38.46	21.74	630–2700	460–3000
WARD 2	(AC)_8_G	47	22	18	77.27	66.67	400–1800	580–2000
ARCADE 3	(CA)_8_TG	49	22	22	45.45	27.27	400–2000	400–1700
**In total**			**131**	**121**				
**Average**					**51.74 ± 7.59**	**48.23 ± 7.23**		

LA—*L. annotinum*, LC—*L. clavatum*.

**Table A2:** Summary of published growth data of two *Lycopodium* species.

Measurement	Result, cm	Reference
***Lycopodium annotinum* L.**		
Total growth/year	44.2	Oinonen [19]
Growth up front/year	22.1	[19]
Average and range of rhizome growth/year	32 (22–45)	[69]
Average number and range of aerial shoots/year	7.9 (7–9)	[69]
Root growth/year	0.7	[70]
Length of horizontal module with 1 root	9.2	[70]
Mean annual extension/year	6.3	[71]
Mean annual extension/year	20	
***Lycopodium clavatum* L.**		
Total growth/year	50.2	[19]
Growth up front/year	25.1	[19]
Average and range of rhizome growth/year	70 (48–103)	[69]
Average number and range of aerial shoots/year	12.8 (12–14)	[69]

**Table A3:** Growth dynamics of young sporophytes of *Lycopodium annotinum* in *Vaccinium myrtillosum* Scots pine stands near Beržupis rivulet, Varėna District *.

Measurement	1985	1987	1989	1991
Number of sprouts	23	25	21	16
New sprouts	-	9	3	-
Died off sprouts	-	7	7	5
Average length of horizontal modules, cm	8 ± 1.5	12.5 ± 1.5	23.3 ± 2.5	33.7 ± 3.4
Maximum length of horizontal modules, cm	11	27	60	113

* Adapted from [38] Naujalis (1995).

**Table A4:** *Lycopodium clavatum* occurrence in Vaccinio-myrtilosa forests in Lithuania *.

Age Group	No. of Plots Evaluated	Overall Coverage, %
26–35	13	Up to 20
36–45	19	Up to 20
46–55	15	Up to 20
66–75	8	Up to 20
76–85	20	Up to 20
86–100	6	41–60
101–120	10	Up to 20
121–140	4	61–80
141>	5	41–60

* Adapted from [56]. Karazija (2003). No *L. clavatum* sporophytes detected in pine stands younger than 26 years.

## References

[1] Eriksson O. (1993). Dynamics of genets in clonal plants. Trends Ecol. Evol..

[2] Honnay O., Bossuyt B. (2005). Prolonged clonal growth: Escape route or route to extinction?. Oikos.

[3] Grime J.P. (2001). Plant Strategies and Vegetation Processes.

[4] Eriksson O. (1989). Seedling dynamics and life histories in clonal plants. Oikos.

[5] Callaghan T.V., Headley A.D., Svensson B.M., Lixian L., Lee J.A., Lindley D.K. (1986). Modular growth and function in the vascular cryptogam *Lycopodium annotinum*. Proc. R. Soc. Lond. Ser. B.

[6] Wittig R., Jungman R., Ballach H.J. (2007). The extent of clonality in large stands of *Lycopodium annotinum* L.. Flora.

[7] Svensson B.M., Rydin H., Carlsson B.A., van der Maarel E., Franklin J. (2013). Clonal plants in the community. Vegetation Ecology.

[8] Bruchmann H. (1898). Über die Prothallien und die Keimpflanzen mehrerer europäischer Lycopodien, und zwar über die von Lycopodium clavatum, L. annotinum, L. complanatum und L. selago.

[9] Pressel S., Bidartondo M.I., Field K.J., Rimington W.R., Duckett J.G. (2016). Pteridophyte fungal associations: Current knowledge and future perspectives. J. Syst. Evol..

[10] Perez-Lamarque B., Laurent-Webb L., Bourceret A., Maillet L., Bik F., Cartier D., Labolle F., Holveck P., Epp D., Selosse M.A. (2023). Fungal microbiomes associated with Lycopodiaceae during ecological succession. Environ. Microbiol. Rep..

[11] Headley A.D., Callaghan T.V., Lee J.A. (1988). Water uptake and movement in the clonal plants, *Lycopodium annotinum* L. and *Diphasiastrum complanatum* (L.) Holub. New Phytol..

[12] Newell S.Y., Carroll G.C., Wicklow D.T. (1992). Estimating fungal biomass and productivity in decomposing litter. The Fungal Community: Its Organization and Role in the Ecosystem.

[13] Landa K., Benner B., Watson M.A., Gartner J. (1992). Physiological integration for carbon in mayapple (*Podophyllum peltatum*), a clonal perennial herb. Oikos.

[14] Bainard J.D., Henry T.A., Bainard L.D., Newmaster S.G. (2011). DNA content variation in monilophytes and lycophytes: Large genomes that are not endopolyploidy. Chromosome Res..

[15] Marchant D.B., Chen G., Cai S., Chen F., Schafran P., Jenkins J., Shu S., Plott C., Webber J., Lovell J.T. (2022). Dynamic genome evolution in a model fern. Nat. Plants.

[16] Eriksson O. (1992). Evolution of seed dispersal and recruitment in clonal plants. Oikos.

[17] Rimgailė-Voicik R., Naujalis J.R. (2022). Techniques for locating and analyzing subterranean *Lycopodium* and *Diphasiastrum* gametophytes in the field. Appl. Plant Sci..

[18] Rimgailė-Voicik R., Naujalis J.R. (2016). Presence of juvenile club moss (Lycopodiaceae) sporophytes and gametophytes in relation to vegetation cover in dry pine forests. Am. Fern J..

[19] Oinonen E. (1968). *Lycopodium clavatum* L. ja *L. annotinum* L.-kasvustojen laajuus rinnastettuna samanpaikkaisiin *L. complanatum* L.-ja *Pteridium aquilinum* (L.) Kuhn—Esiintymiiin sekä puuston ikään j paloaikoihin. Acta For. Fenn..

[20] Watson M.A., Casper B.B. (1984). Morphogenetic constraints on patterns of carbon distribution in plants. Annu. Rev. Ecol. Evol. Syst..

[21] Yang X., Poelmans W., Grones C., Lakehal A., Pevernagie J., Van Bel M., Njo M., Xu L., Nelissen H., De Rybel B. (2023). Spatial transcriptomics of a lycophyte root sheds light on root evolution. Curr. Biol..

[22] Hetherington A.J., Dolan L. (2017). The evolution of lycopsid rooting structures: Conservatism and disparity. New Phytol..

[23] Sutherland W.J., Stillman R.A. (1988). The foraging tactics of plants. Oikos.

[24] Horn K., Franke T., Unterseher M., Schnittler M., Beenken L. (2013). Morphological and molecular analyses of fungal endophytes of achlorophyllous gametophytes of *Diphasiastrum alpinum* (Lycopodiaceae). Am. J. Bot..

[25] Eames A.J. (1942). Illustrations of some *Lycopodium* gametophytes. Am. Fern J..

[26] Chytrý M., Tichý L., Hennekens S.M., Knollová I., Janssen J.A.M., Rodwell J.S., Peterka T., Marceno C., Landucci F., Danihelka J. (2020). EUNIS Habitat Classification: Expert system, characteristic species combinations and distribution maps of European habitats. Appl. Veg. Sci..

[27] European Council (1992). Council Directive 92/43/EEC of 21 May 1992 on the conservation of natural habitats and of wild fauna and flora. Off. J. Eur. Union.

[28] IUCN (2017). *Lycopodium* *annotinum*. https://www.iucnredlist.org/species/18821015/85433421#assessment-information.

[29] Commission Regulation (EC) No 318/2008 of 31 March 2008, Amending Council Regulation (EC) No 338/97 on the Protection of Species of Wild Fauna and Flora by Regulating Trade Therein. https://eur-lex.europa.eu/LexUriServ/LexUriServ.do?uri=OJ:L:2008:095:0003:0062:EN:PDF.

[30] Braun-Blanquet J. (1928). Pflanzensoziologie: Grundzüge der Vegetationskunde.

[31] Ellenberg H. (1974). Zeigerwerte der Gefaesspflanzen Mitteleuropas. Scripta Geobotanica IX.

[32] Tichý L., Axmanová T., Dengler J., Guarino R., Jansen F., Midolo G., Nobis M.P., Van Meerbeek K., Acic S., Attore F. (2023). Ellenberg-type indicator values for European vascular plant species. J. Veg. Sci..

[33] Pec G.J., Karst J., Sywenky A.N., Cigan P.W., Erbilgin N., Simard S.W., Cahill J.F. (2015). Rapid Increases in Forest Understory Diversity and Productivity following a Mountain Pine Beetle (*Dendroctonus ponderosae*) Outbreak in Pine Forests. PLoS ONE.

[34] Gilliam F.S. (2007). The ecological significance of the herbaceous layer in temperate forest ecosystems. BioScience.

[35] Cherubini P., Battipaglia G., Innes J.L. (2021). Tree vitality and forest health: Can tree-ring stable isotopes be used as indicators?. Curr. For. Rep..

[36] Hooper D.U., Adair E.C., Cardinale B.J., Byrnes J.E.K., Hungate B.A., Matulich K.L., Gonzalez A., Duffy J.E., Gamfeldt L., O’Connoret M.I. (2012). A global synthesis reveals biodiversity loss as a major driver of ecosystem change. Nature.

[37] Lithuanian Forest Cutting Rules. https://e-seimas.lrs.lt/portal/legalAct/lt/TAD/TAIS.364764/asr.

[38] Naujalis J.R. (1995). Sporiniai Induočiai Kaip Augalų Bendrijų Komponentai.

[39] Oinonen E. (1971). The time table of vegetative spreading in oak fern (*Carpogymnia dryopteris* (L.) Löve & Löve) and may-lily (*Maianthemum bifolium* (L.) F. W. Schmidt in Southern Finland. Acta For. Fenn..

[40] Oinonen E. (1967). Keltalieon (*Lycopodium complanatum* L.) itiöllinen uudistuminen Etelä-Suomessa kloonien laajuutta ja ikää koskevan tutkimuksen valossa. Acta For. Fenn..

[41] Nemchinova A.V., Krinycyn I.G., Smirnova L.A. (2014). Biometricheskye osobennosti biomorf plauna bulavovidnovo (*Lycopodium clavatum* L.) v ontogeneze. Vestnik KGU Im. N. A. Nekrasova.

[42] Svensson B.M., Floderus B., Callaghan T.V. (1994). *Lycopodium annotinum* and light quality: Growth responses under canopies of two *Vaccinium* species. Folia Geobot..

[43] Moola F.M., Vasseur L. (2004). Recovery of late-seral vascular plants in a chronosequence of post-clearcut forest stands in coastal Nova Scotia, Canada. Plant Ecol..

[44] Harper J.L. (1977). Population Biology of Plants.

[45] Levin D.A., Crepet W.L. (1973). Genetic variation in *Lycopodium lucidulum*: A phylogenetic relic. Evolution.

[46] Ellstrand N.C., Roose M.L. (1987). Patterns of Genotypic Diversity in Clonal Plant Species. Am. J. Bot..

[47] Reisch C., Poschlod P. (2004). Clonal diversity and subpopulation structure in central European relict populations of *Saxifraga paniculata* Mill. (Saxifragaceae). Feddes Repert..

[48] Ranker T.A., Geiger J.M.O., Ranker T.A., Haufler C.H. (2008). Population genetics. Biology and Evolution of Ferns and Lycophytes.

[49] Schnittler M., Horn K., Kaufmann R., Rimgailė-Voicik R., Klahr A., Bog M., Fuchs J., Bennert H.W. (2019). Genetic diversity and hybrid formation in Central European club-mosses (*Diphasiastrum*, Lycopodiaceae)—New insights from cp microsatellites, two nuclear markers and AFLP. Mol. Phylogenet. Evol..

[50] Shorohova E., Kneeshaw D., Kuuluvainen T., Gauthier S. (2011). Variability and dynamics of old-growth forests in the circumboreal zone: Implications for conservation, restoration and management. Silva Fenn..

[51] State Forest Service (2021). Forest Statistic. https://amvmt.lrv.lt/lt/atviri-duomenys-1/misku-statistikos-leidiniai/misku-ukio-statistika/2021-m-1.

[52] Marozas V., Sasnauskienė J. (2021). Changes of ground vegetation after shelter wood cuttings in pine forests, the hemiboreal zone, Lithuania. Balt. For..

[53] Herben T., Chytrý M., Klimešová J. (2016). A quest for species-level indicator values for disturbance. J. Veg. Sci..

[54] Sokołowski A.W., Paluch R. (2006). The effect of clear cutting and artificial regeneration with pine on species composition of fresh coniferous stands in the Białowieża Primeval Forest. Sylwan.

[55] Obidziński A. (2001). Disturbance as an element of forest dynamics. Sylwan.

[56] Karazija S. (2003). Age related dynamics of pine forest communities in Lithuania. Balt. For..

[57] Jogiste K., Frelich L.E., Laarmann D., Vodde F., Baders E., Donis J., Jansons A., Kangur A., Korjus H., Koster K. (2018). Imprints of management history on hemiboreal forest ecosystems in the Baltic States. Ecosphere.

[58] National Land Service under the Ministry of Agriculture of the Republic of Lithuania (2016). Lithuanian National Atlas.

[59] State Forest Service under the Lithuanian Ministry of Environment (2023). Forest Cadastre Data. https://www.geoportal.lt/map#portalAction=openService&serviceUrl=https%3A%2F%2Fwww.geoportal.lt%2Fmapproxy%2Fvmt_mkd%2FmapServer.

[60] Nobis M., Hunziker U. (2005). Automatic thresholding for hemispherical canopy photographs based on edge detection. Agric. For. Meteorol..

[61] Frazer G.W., Canham C.D., Lertzman K.P. (1999). Gap Light Analyzer (GLA): Imaging Software to Extract Canopy Structure and Gap Light Transmission Indices from True-Colour Fish Eye Photographs.

[62] Kembel S.W., Cowan P.D., Helmus M.R., Cornwell W.K. (2010). Picante: R tools for integrating phylogenies and ecology. Bioinformatics.

[63] Nei M. (1978). Estimation of average heterozigosity and genetic distance from a small number of individuals. Genetics.

[64] Peakall R., Smouse P. (2006). GenAlEx v.6: Genetic analysis in Excel. Population genetic software for teaching and research—An update. Bioinformatics.

[65] McKinney W. Data Structures for Statistical Computing in Python. Proceedings of the 9th Python in Science Conference.

[66] Vallat R. (2018). Pingouin: Statistics in Python. J. Open Source Softw..

[67] Doyle J.J., Doyle J.L. (1990). Isolation of plant DNA from fresh tissue. Focus.

[68] Patamsytė J., Čėsnienė T., Naugžemys D., Kleizaitė V., Vaitkūnienė V., Rančelis V., Žvingila D. (1990). Genetic diversity of warty cabbage (*Bunias orientalis* L.) revealed by RAPD and ISSR markers. Zemdirbyste.

[69] Primack R.B. (1973). Growth patterns of five species of *Lycopodium*. Am. Fern J..

[70] Callaghan T.V., Svensson B.M., Headley A.D. (1986). Modular growth of *Lycopodium annotinum*. Fern Gaz..

[71] Svensson B.M. (1987). Studies of the Metapopulation Dynamics of *Lycopodium annotinum* and Its Microenvironment. Ph.D. Thesis.

